# Investigating the cardiorespiratory fitness gene *COX7A2L* in cardiomyocytes: Viability and mitochondrial function

**DOI:** 10.1371/journal.pone.0326249

**Published:** 2025-06-25

**Authors:** Ada Nilsen Nordeidet, Gurdeep S. A. Marwarha, Øystein Røsand, Victoria Johansen, Karin Garten, Morten A. Høydal, Mette Langaas, Anja Bye

**Affiliations:** 1 Cardiac Exercise Research Group (CERG), Department of Circulation and Medical Imaging, Faculty of Medicine and Health Sciences, Norwegian University of Science and Technology (NTNU), Trondheim, Norway; 2 Group of Molecular and Cellular Cardiology, Department of Circulation and Medical Imaging, Faculty of Medicine and Health, Norwegian University of Science and Technology (NTNU), Trondheim, Norway; 3 Department of Circulation and Medical Imaging, Faculty of Medicine and Health, Norwegian University of Science and Technology, Trondheim, Norway; 4 Department of Mathematical Sciences, Faculty of Information Technology and Electrical Engineering, Norwegian University of Science and Technology (NTNU), Trondheim, Norway; 5 Department of Cardiology, St. Olavs Hospital, Trondheim University Hospital, Trondheim, Norway; University of Tennessee Health Science Center College of Medicine Memphis, UNITED STATES OF AMERICA

## Abstract

Low cardiorespiratory fitness (CRF) is a well-established risk factor for cardiovascular disease (CVD) and all-cause mortality. Since CRF is largely genetically determined, understanding the genetic influences on CRF might reveal the protective mechanisms of high CRF. One gene found to be associated with CRF is *COX7A2L*. COX7A2L is a mitochondrial supercomplex assembly factor, but its role in cellular metabolism remains a topic of discussion. We hypothesized that COX7A2L could play a role in cellular respiration in cardiomyocytes, affecting cardiac function and CRF. To determine the effect of COX7A2L on cardiomyocyte function, we overexpressed and knocked down *COX7A2L* in human AC16 cardiomyocytes and performed MTT assays and Seahorse XF Cell Mito Stress Tests to assess cell viability and mitochondrial function. For the mitochondrial function measurements, we stimulated the cells with isoproterenol to investigate if the effect of altering *COX7A2L* levels would be larger under simulated increased energy demand. Overexpression and knockdown were validated using sandwich ELISA. Our findings showed that altering *COX7A2L* expression in human AC16 cardiomyocytes did not significantly affect cell viability or mitochondrial function. Further research is necessary to determine whether COX7A2L influences cardiomyocyte function and CRF.

## Introduction

Cardiorespiratory fitness (CRF) is a major predictor of future cardiovascular health, and higher CRF levels are associated with a lower risk of cardiovascular disease (CVD) and mortality [1,2]. CRF is a heritable, polygenic trait, with genetic factors estimated to explain around 60% of individual variation [3]. Despite this, the specific genes involved and how they contribute to the protective effects of CRF remain poorly understood [4].

Recent findings have highlighted the gene *COX7A2L* as a genetic determinant of CRF. Benegiamo et al. (2022) demonstrated that genetic variation in *COX7A2L* influences CRF in both mice and humans through modulation of mitochondrial respiration [5]. Increased *COX7A2L* expression enhanced respiration, particularly in skeletal muscle. Another study found that mice with reduced Cox7a2l levels in the mitochondria of the heart had lower exercise performance, suggesting a possible functional role for COX7A2L in the heart as well [6].

Because cardiac output is the primary limiting factor of CRF and that the heart is a highly energy-demanding organ, understanding how COX7A2L influences mitochondrial energy production in cardiomyocytes (CMs) is of critical interest. CMs rely heavily on mitochondrial ATP to support their continuous contractile activity. Within the mitochondrial inner membrane, respiratory chain complexes organize into larger structures known as supercomplexes (SCs), which are suggested to enhance electron transport efficiency. Two SC formations are CIII + CIV and CI+CIII+CIV, the latter known as the respirasome [7–9]. COX7A2L, also known as super complex assembly factor 1 (SCAF1), facilitates the assembly of respiratory complex III (CIII) and IV (CIV), thereby contributing to the formation of SCs [10,11]. SC assembly is dynamic and organizes electron flux to optimize the use of available substrates [10], and the assembly including the role of assembly factors might be dependent on the tissue type and metabolic status of the cell [11,12]. The functional importance of SCs in human cells is not well-established [13,14], including for energy-demanding tissue like the heart. However, the compromised assembly of SCs in CVDs [15,16] strongly indicates their importance in cardiac health. In human non-cardiac systems, COX7A2L has been shown to promote SC-assembly and increase cellular respiration and metabolic efficiency [17,18]. Similar findings have also been reported in zebrafish [19]. However, the role of COX7A2L in CMs remains unexplored, and its role in cellular metabolism is still under debate [20,21].

Because CM contraction depends on mitochondrial ATP production, any disruption in SC assembly that impairs oxidative phosphorylation could weaken contractile function and, in turn, reduce cardiac output and CRF. Additional aspects of CM physiology that relies on efficient mitochondrial energy production are calcium handling, electrical stability, redox balance, metabolic flexibility, and sell survival – functions that are essential for maintaining optimal cardiac performance and, by extension, high CRF [22,23]. Based on this, we hypothesize that COX7A2L influences CM function through its regulation of SC assembly and mitochondrial function. Elucidating the role of COX7A2L in CMs may offer new insights into the molecular mechanisms that connect high CRF with improved cardiovascular health. In this study, we investigated the role of *COX7A2L* in mitochondrial function and cell viability in human-derived AC16 CMs. We hypothesized that knockdown of *COX7A2L* expression would impair cell function parameters, and that overexpression would have the opposite effect. To our knowledge, this is the first *in vitro* study investigating the potential role of COX7A2L in CMs.

## Materials and methods

### Cell culture

Immortalized human AC16 CMs (EMD Millipore/Merck Millipore/Sigma-Aldrich, #SCC109, Darmstadt, Germany) [24] were cultured in 100 mm cell culture treated dishes (Thermo Fisher Scientific, #130182, Waltham, MA, USA) with Dulbecco’s modified Eagle’s medium (DMEM)/F-12 with 2 mM glutamine (Thermo Fisher Scientific, #11330057), 12.5% fetal bovine serum (Sigma Aldrich, #F7524, Burlington, MA, USA) and, 1% antibiotic-antimycotic solution (Merck Life Sciences/Sigma Aldrich, #A5955, Burlington, MA, USA). The cells were cultured at 37°C in a humidified incubator with 5% CO_2_. Before the transfection, the cell passage number was between 6 and 9.

### Transfection

For the overexpression experiments, the cells were reverse transfected with either the pReceiver-M12 expression vector encoding the COX7A2L gene (GeneCopoeiaTM, #T7055, Rockville, MD, USA), or its corresponding empty vector. For the knockdown experiments, the cells were reverse transfected with either the TRC short hairpin RNA (shRNA) vector (Horizon, #RHS4533-EG9167, Cambridge, UK) or the pLKO.1 empty vector (Horizon, #RHS4080, Cambridge, UK). The shRNA construct was designed to target *COX7A2L* and was cloned into lentiviral vectors. The transfection was performed using PolyFect Transfection Reagent (Qiagen, #301107, Venlo, Netherlands) in accordance with the manufacturer’s protocol. The plasmid load was 1 µg per 1.2 x 10^6^ cells.

### Cell viability assay

Cell viability was determined using the MTT cell proliferation assay (Cell Proliferation Kit I, Roche, #11465007001, Basel, Switzerland). Cells were transfected as previously described and seeded at a density of 1.5 x 10^4^ cells per well in a 96-well plate, including four biological replicates and six technical replicates. The remaining cells were plated back to 100 mm plates for knockdown and overexpression validation purposes. The culture medium was changed after 6 hours and then again after 16 hours. After 24 hours, 20 µL MTT reagent was added to each well. MTT was also added to the wells containing only medium to serve as blank negative controls. At this point, 46 hours had elapsed since the transfection mixture was added to the cells. Four hours later, 100 µL solubilization buffer was added to each well. After overnight incubation at 37°C, the absorbance was measured at 580 nm using the Varioskan LUX microplate reader (Thermo Fisher Scientific, #VLBLATGD2). The experiment was repeated to ensure reproducibility.

### Assessing mitochondrial function

To assess mitochondrial function, we used the Seahorse Cell Mito Stress Test (Agilent, # 103015−100, Santa Clara, CA, USA). Oxygen consumption rates (OCR) of the cells were measured directly using the Seahorse XF Pro analyzer. Seahorse XF Pro Cell culture microplates (Agilent, # 103793−100) were treated with Cell Tak (22.4 µg/mL) (Corning, #CB-40240, Corning, NY, USA) and cells were transfected as previously described and seeded onto the plate at a density of 1 x 10^4^ cells per well in 80 µL growth medium. The remaining cells were plated back to 100 mm plates for knockdown and overexpression validation purposes. The day after, the culture medium was changed. Twenty-four hours after this, and 1 hour prior to the assay, the cells were washed twice with XF DMEM assay medium (Agilent, #103575−100) and then incubated with this medium for 1 hour in a non-CO_2_ incubator. The assay medium was supplemented with 0.5 mM pyruvate (Agilent, #103578−100), 2.5 mM glutamine (Agilent, #103579−100), and 17.5 mM glucose (Agilent, #103577−100). The cells were treated with 10 µM isoproterenol (ISO) (Sigma, #I5627-25G, Saint-Louis, MO, USA), a β-adrenergic receptor agonist, for adrenergic stimulation of the cells that simulates increased energy demand. We performed serial dilution treatments with different ISO concentrations to test efficiency. The OCR was measured over a period of around 80 minutes, during which sequential injections of oligomycin, FCCP, and antimycin A + rotenone were performed at final concentrations of 1.5 µM, 1 µM and 0.9 µM, respectively. Since the transfected cells were allowed to grow in the Seahorse XF 96 well plate for two days prior to measuring mitochondrial function, it was crucial to normalize the OCR data. We employed the method of nuclei staining and direct cell count, which is considered the gold standard. We stained cell nuclei in all wells with 2 µg/mL Hoechst stain (Thermo Fisher Scientific, #62249) and counted them using the EVOS FL Auto Cell Imaging System (Thermo Scientific, #AMF5000SV), and the CellProfiler software [25]. Agilent Seahorse Analytics Software was used to obtain data on all parameters, including basal respiration, maximal respiration, and spare respiratory capacity (SRC). All parameters were calculated according to the manufacturer’s guidelines [26]. The experiment was repeated to ensure reproducibility.

### Validation of knockdown and overexpression

To validate the knockdown and overexpression of *COX7A2L*, we used sandwich ELISA to determine the amount of COX7A2L in the harvested cell lysates. First, the protein concentration of each sample was measured with the Bradford assay (Pierce Coomassie Plus Bradford assay, Thermo Scientific, #23236). 10 ng of the respective capture antibodies were immobilized in each well of the 96-well microplate. The lysates were incubated with the immobilized capture antibodies overnight at 4°C (30 µg protein per well). The 96-well microplate wells were washed with TBS-T three times over 15 minutes, then subsequently incubated with the respective detection antibodies overnight at 4°C. The 96-well microplate wells were washed with TBS-T six times over 45 minutes. Immunodetection followed next. The 96-well microplate wells were incubated with the alkaline phosphatase (AP)-conjugated secondary antibody for 2 hours. Then, the AP substrate PNPP was added as a chromophore for the colorimetric read-out at 470 nm. The specificity of the antibody signal was established through peptide-blocking assays, using antibody-blocking peptides corresponding to the specific epitopes for the detection antibodies. The absorbances from the peptide-blocking assays were used for experimental blank correction. The blank-corrected values were then normalized and expressed as signals relative to experimental control. A complete list of all antibodies, reagents, and buffers used can be found in the supplementary S1 File.

### Statistical analysis

Differences between experimental groups in the MTT assay were investigated using independent two sample t-tests. The analyses were performed using GraphPad Prism (version 10.1.2). To analyze the effects of *COX7A2L* knockdown, *COX7A2L* overexpression, and ISO treatment in the Seahorse assays, we fitted a linear mixed-effect model with knockdown/overexpression treatment and ISO treatment as fixed factors (with interaction), and with experiment as random intercept. These analyses were performed using R [27]. A p-value below 0.05 (p < 0.05) was considered significant. Quantitative data from all assays are presented as descriptive mean values ± SD.

## Results

**Fig 1:**
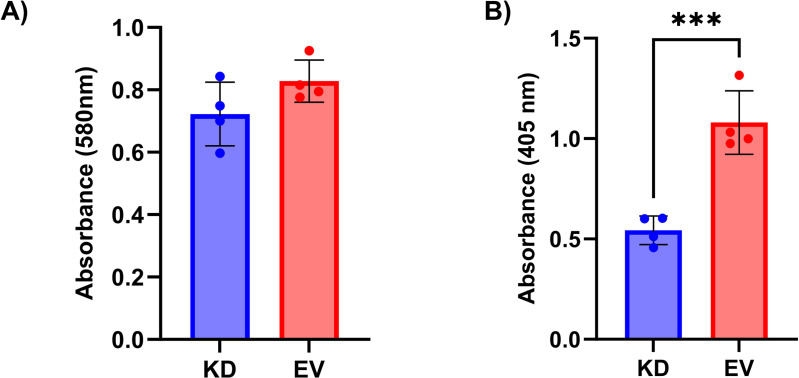
*COX7A2L* knockdown did not affect cell viability. (A) Our results showed that knockdown of *COX7A2L* has no significant effect (p = 0.13) on cell viability measured with the MTT assay. (B) Validation of *COX7A2L* knockdown using sandwich ELISA. The amount of COX7A2L is quantified indirectly by measuring the absorbance of the ELISA enzyme substrate. COX7A2L was significantly knocked down (p = 0.0008). Data from the MTT assay were analyzed using an independent two sample t-test and are expressed as mean values ± SD (N = 4). KD = knockdown, EV = empty vector.

**Fig 2:**
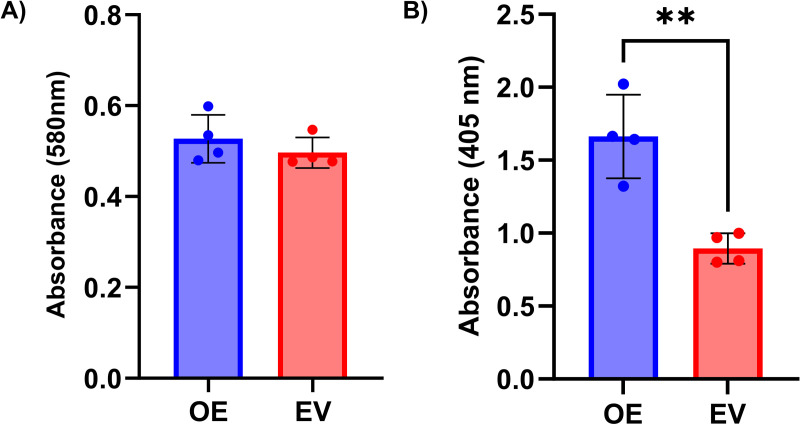
*COX7A2L* overexpression did not affect cell viability. (A) Our results showed that overexpression of *COX7A2L* has no significant effect (p = 0.37) on cell viability measured with the MTT assay. (B) Validation of *COX7A2L* overexpression using sandwich ELISA. The amount of COX7A2L is quantified indirectly by measuring the absorbance of the ELISA enzyme substrate. COX7A2L was significantly overexpressed (p = 0.002). Data from the MTT assay were analyzed using an independent two sample t-test and are expressed as mean values ± SD (N = 4). OE = overexpression, EV = empty vector.

To assess whether altering the expression of *COX7A2L* affects cell viability, we performed an MTT assay following overexpression or knockdown of *COX7A2L* in AC16 CMs. We found that neither knockdown nor overexpression of *COX7A2L* affects cell viability in AC16 CMs (t(6) = 1.72, p = 0.13; Fig 1A, t(6) = 0.98, p = 0.37; Fig 2A). ELISA confirmed successful knockdown (EV: 1.08 ± 0.16 AU vs. KD: 0.54 ± 0.07 AU; p = 0.0008; Fig 1B) and overexpression (EV: 0.89 ± 0.1 AU vs. OE: 1.66 ± 0.29 AU; p = 0.002; Fig 2B). Both experiments were repeated, and they also showed no effect of altered *COX7A2L* expression.

**Fig 3:**
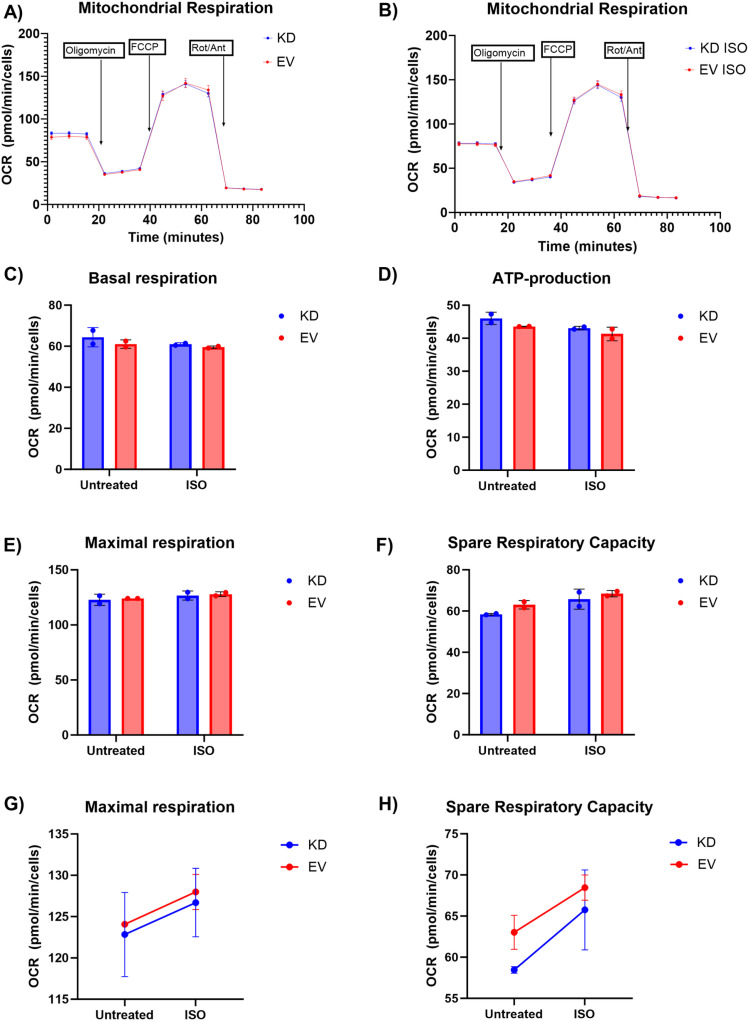
*COX7A2L* knockdown did not affect mitochondrial function. Our results showed that *COX7A2L* knockdown had no significant effect on mitochondrial respiration rates, either under basal conditions (A) or when stimulated with isoproterenol (ISO) (B). The knockdown had no significant effects on basal respiration (C), ATP production (D), maximal respiration (E and G), or spare respiratory capacity (SRC) (F and H). ISO had a weak non-significant effect on maximal respiration and SRC (G and H). KD = knockdown, EV = empty vector, OCR = oxygen consumption rate, Rot/Ant = rotenone and antimycin A.

To assess whether *COX7A2L* knockdown affects mitochondrial function, we used the Seahorse XF96 Cell Mito Stress test and measured the OCR in real time. The results showed that knockdown of *COX7A2L* did not have a significant effect on mitochondrial respiration rates, either under basal conditions (Fig 3A) or when stimulated with ISO (Fig 3B). The estimates from the linear mixed effect model show that *COX7A2L* knockdown did not have a significant effect on basal respiration (Fig 3C), ATP production (Fig 3D), maximal respiration (Fig 3E) or spare respiratory capacity (SRC). The weak and non-significant effect on maximal respiration and SRC was negative (Fig 3E–H). ISO had a weak non-significant positive effect on both maximal respiration and SRC (Fig 3G, H). When comparing the groups treated with ISO, knockdown had a negative non-significant effect on maximal respiration and SRC (Fig 3E–H). The results from the statistical analyses can be found in the supplementary S2 Table.

**Fig 4:**
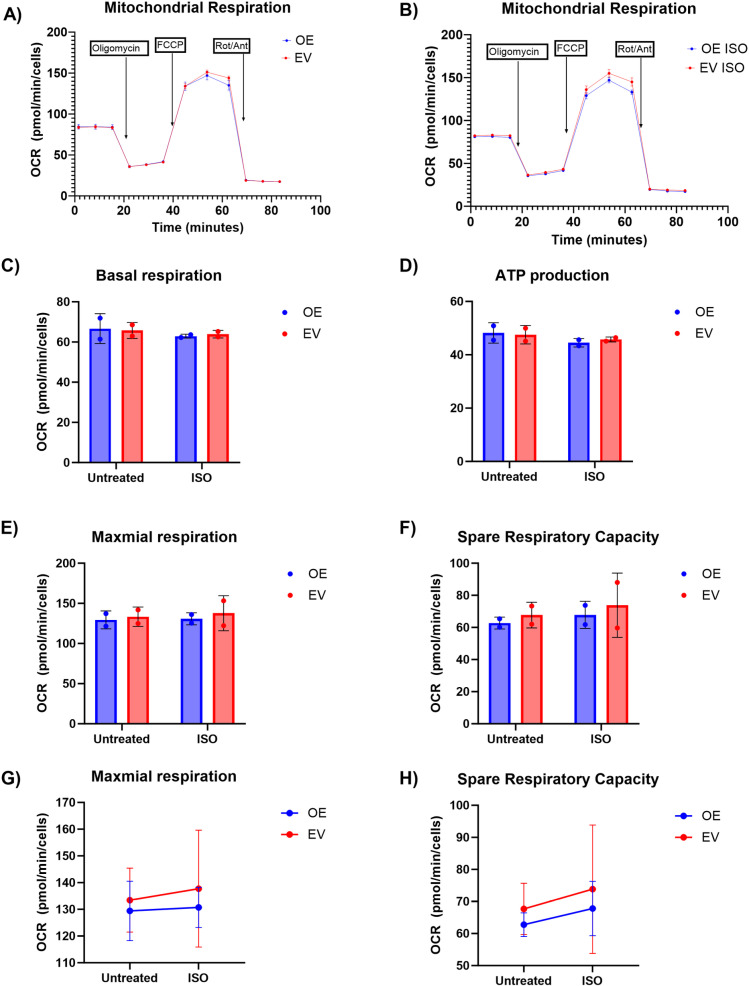
*COX7A2L* overexpression did not affect mitochondrial function. Overexpression of *COX7A2L* had no significant effect on mitochondrial respiration rates, either under basal conditions (A) or when stimulated with isoproterenol (ISO) (B). Overexpression had no significant effects on basal respiration (C), ATP production (D), maximal respiration (E and G), or spare respiratory capacity (SRC) (F and H). ISO had a weak non-significant effect on maximal respiration and SRC (G and H). OE = overexpression, EV = empty vector, OCR = oxygen consumption rate, Rot/Ant = rotenone and antimycin A.

We also wanted to assess whether *COX7A2L* overexpression affects mitochondrial function. The results showed that overexpression of *COX7A2L* did not have a significant effect on mitochondrial respiration rates, either under basal conditions (Fig 4A) or when stimulated with ISO (Fig 4B). The estimates from the linear mixed effect model show that *COX7A2L* overexpression did not have a significant effect on basal respiration (Fig 4C), ATP production (Fig 4D), maximal respiration (Fig 4E) or SRC (Fig 4F). The weak and non-significant effect on maximal respiration and SRC was negative (Fig 4E–H). ISO had a weak non-significant positive effect on both maximal respiration and SRC (Fig 4G, H). When comparing the groups treated with ISO, overexpression had a negative non-significant effect on maximal respiration and SRC (Fig 4E, F). The results from the statistical analysis can be found in the supplementary S3 Table.

## Discussion

Investigating the effect of CRF genes in the heart is important for understanding the mechanisms underlying the cardioprotective effects of high CRF. In this study, we tested whether altering expression of the CRF gene *COX7A2L* in AC16 CMs would affect cell viability and mitochondrial activity. Our approach was motivated by previous research linking COX7A2L to the assembly of mitochondrial respiratory SCs and enhanced cellular respiration. We hypothesized that altering *COX7A2L* expression would impact mitochondrial function in CMs, given the heart’s high metabolic demand and reliance on efficient cellular respiration. Our further hypothesis was that if altered *COX7A2L* expression substantially disrupted the mitochondrial respiratory chain, this would negatively affect the cell viability.

Contrary to our hypothesis, neither overexpression nor knockdown of *COX7A2L* significantly impacted cell viability or mitochondrial respiration. Using the MTT cell proliferation assay, we found that *COX7A2L* knockdown did not reduce CM viability compared to controls with empty vectors. Overexpression of *COX7A2L* also had no effect on CM viability. As a robustness analysis, we performed a non-parametric Mann-Whitney U test, and it showed the same results. Our results from the mitochondrial function assessments showed that altering the level of COX7A2L did not significantly affect mitochondrial respiration parameters, including basal respiration, ATP production, maximal respiration, or SRC. Maximal respiration is the highest rate of respiration that a cell can achieve, and SRC indicates the cell’s capacity to respond to increased energy demand, serving as an indicator of cell fitness [28]. We considered these key parameters, as *COX7A2L* is a CRF-associated gene. The estimates showed that both overexpression and knockdown had a weak negative effect on maximal respiration and SRC, but since the effects are non-significant, we should be careful interpreting their direction. We hypothesized that increased *COX7A2L* expression would increase cellular respiration, through increased assembly of SCs and possibly respirasomes, and that the opposite would occur with knock down. However, our findings indicate that COX7A2L does not play a role in cellular respiration in AC16 CMs.

One possible explanation for these negative results is cell-type specificity. The functional relevance of SCs is not well established, and the formation of SCs in mitochondria might be more important in certain tissues, organs, or cells [8]. While COX7A2L has been shown to promote SC-assembly and enhance respiration in other human cell types, like skeletal muscle, its functional role in CMs may be less pronounced or compensated by other mechanisms. Also, two studies using human cell lines have reported that COX7A2L was involved in the stabilization and organization of SCs without affecting mitochondrial bioenergetics or respirasome formation [20,21]. For future directions, it would be interesting to directly evaluate SC and respirasome assembly by quantifying the different constellations of mitochondrial chain complexes and associated proteins as it remains unclear whether altering *COX7A2L* expression affects SC/respirasome formation.

Another consideration is the experimental context. Under basal culture conditions, AC16 CMs are likely not operating at maximal energy capacity, and thus alterations in *COX7A2L* may not produce discernible effects on mitochondrial function or cell viability. To address this, we exposed cells to ISO for an acute beta-adrenergic activation and elevated energy demand. A previous study on a mouse CM cell line showed that ISO treatment (10 µM, 1h) increased respiration [29] and multiple studies on AC16 CMs employ ISO to mimic persistent beta-adrenergic stimulation inducing disease phenotypes [30–32]. While ISO treatment led to a modest increase in mitochondrial respiration, the effect did not reach statistical significance. This outcome indicates insufficient acute effects in AC16 CMs using the specified dose and duration. Activation of beta-adrenergic receptors can affect mitochondria in various ways depending on the type of CM and the specific context [33]. The outcome could also indicate that COX7A2L is not a limiting factor for mitochondrial adaptation to acute stress in this cell model. Although COX7A2L expression is shown to be upregulated in response to certain stressors, such as hypoxia, nutrient stress, and ER stress [17,34–36], its role under basal or moderate stress conditions may be limited. This raises the possibility that COX7A2L becomes functionally relevant only under specific physiological or pathological stresses that were not induced in our experiments and should be tried induced in future studies.

### Considerations

Several limitations should be acknowledged. The use of AC16 cells may not capture the full complexity of primary CM physiology. Several previous studies have used AC16 CMs to assess mitochondrial function in CMs [37–39]; however, the utility of AC16 CMs for investigating features relevant for mature cardiac muscle is an open question [40]. Moreover, relatively low *COX7A2L* expression levels in AC16-cells compared to adult heart tissue *in vivo* could explain the lack of effect from knockdown. Future studies should consider using primary CMs or iPSC CMs. Our study could be strengthened by performing additional validation of knockdown and overexpression at the transcriptional level and assessing COX7A2L protein levels specifically in mitochondria.

## Conclusion

In this study we sought to investigate the effect of altered expression of *COX*7A2L, a gene associated with CRF, on CM function. In summary, our data suggests that COX7A2L is not essential for mitochondrial function or viability in AC16 CMs, at least not under the conditions tested. Further research is warranted to determine the potential role of cardiac COX7A2L and to delineate the potential contexts in which COX7A2L becomes functionally significant for cardiac function and CRF.

## Supporting information

S1 File Supplementary materials, including S1 Table. Available at https://doi.org/10.6084/m9.figshare.27721089.v1.
(DOCX)

S2 File S2 Table and S3 Table. Supplementary table 2 and 3. Available at https://doi.org/10.6084/m9.figshare.27721197.v1.
(XLSX)
