# Supplementary material for: Investigating the cardiorespiratory fitness gene COX7A2L in cardiomyocytes: Viability and mitochondrial function
Source: PLoS One. 2025 Jun 25;20(6):e0326249. doi: 10.1371/journal.pone.0326249 (PMC12194157; doi:10.1371/journal.pone.0326249)
Supplement: S1 File — Available at https://doi.org/10.6084/m9.figshare.27721089.v1. (DOCX) [file pone.0326249.s001.docx]

**Supplementary Table 1:** List of monoclonal and polyclonal antibodies used in the ELISA immunoassays.

| Antibody | Application | Amount | Host | Manufacturer | Catalogue # | Resource Identifier ID (RRID)* |
| --- | --- | --- | --- | --- | --- | --- |
| β-Actin | ELISA *capture* | 10 ng/well | Mouse | Santa Cruz Biotechnology | sc-47778 | AB_2714189 |
| β-Actin | ELISA *detection* | 10 ng/well | Rabbit | Cell Signalling Technology | 4970 | AB_2223172 |
| β-Actin antibody blocking peptide | ELISA *detection* | N/A | N/A | Cell Signalling Technology | 1025 | N/A |
| COX7A2L | ELISA *capture* | 10 ng/well | Mouse | Thermo Fisher Scientific | H00009167-B02P | AB_2085707 |
| COX7A2L | ELISA *detection* | 10 ng/well | Rabbit | Novus Biologicals | NBP2-56202 | N/A |
| COX7A2L antibody blocking peptide | ELISA *detection* | N/A | N/A | Novus Biologicals | NBP2-56202PEP | N/A |
| Goat Anti-Rabbit IgG-AP Conjugate | ELISA *detection* | 1:20000 **^€^** | Goat | Sigma Aldrich/ Merck Life Science | A3687 | AB_258103 |

***** RRID Portal – Research Resource Identification Portal

**N/A:** Not Available / Not Applicable

**€:** Amount of secondary antibody cannot be determined as the commercial vendor does not provide the antibody concentration

**List of Reagents and Buffers used in the ELISA immunoassays:**

**PNPP:** p-Nitrophenyl Phosphate, disodium salt (Thermo Fisher Scientific, Oslo, Norway, Catalogue # 37621)

***Non-denaturing* lysis buffer:** 20 mM Tris, 137 mM Nacl, 2 mM EDTA, 1% Nonidet P-40, 10% glycerol, pH 7.4

**TBS-T:** Tris-buffered saline with 0.1% v/v Tween-20 (20 mM Tris, 150 mM Nacl, 0.1% v/v Tween-20)
